# *Gynura procumbens* ethanol extract improves vascular dysfunction by suppressing inflammation in postmenopausal rats fed a high-fat diet

**DOI:** 10.1080/13880209.2021.1970199

**Published:** 2021-09-07

**Authors:** Khuzaidatul Azidah Ahmad Nazri, Qodriyah Haji Mohd Saad, Norsyahida Mohd Fauzi, Fhataheya Buang, Ibrahim Jantan, Zakiah Jubri

**Affiliations:** a Department of Biochemistry, Faculty of Medicine, Universiti Kebangsaan Malaysia Medical Centre (UKMMC), Kuala Lumpur, Malaysia; b Department of Pharmacology, Faculty of Medicine, Universiti Kebangsaan Malaysia Medical Centre (UKMMC), Kuala Lumpur, Malaysia; c Drug and Herbal Research Centre, Faculty of Pharmacy, Universiti Kebangsaan Malaysia, Kuala Lumpur, Malaysia; d Institute of Systems Biology (INBIOSIS), Universiti Kebangsaan Malaysia, UKM Bangi, Bangi, Malaysia

**Keywords:** Blood pressure, ovariectomized, vasorelaxation, vasoconstriction, cholesterol, interleukin-6, tumour necrosis factor-alpha, C-reactive protein

## Abstract

**Context:**

*Gynura procumbens* (Lour.) Merr. (Asteraceae) has been reported to have various pharmacological activities including anti-inflammatory effects.

**Objective:**

This study sought to determine whether *Gynura procumbens* (GP) could improve vascular reactivity by suppressing inflammation in postmenopausal rats fed with five-times heated palm oil (5HPO) diet.

**Materials and methods:**

Forty-eight female Sprague-Dawley rats were randomly divided into sham [non-ovariectomized; grouped as control, GP extracts (250 and 500 mg/kg), atorvastatin (ATV, 10 mg/kg)] and postmenopausal (PM) groups [ovariectomized rats fed with 5HPO; grouped as PM, GP extracts (250 and 500 mg/kg) and ATV (10 mg/kg)]. Each group (*n* = 6) was either supplemented with GP extract or ATV orally once daily for 6 months.

**Results:**

In comparison with the untreated PM group, 250 and 500 mg/kg GP supplementation to PM groups reduced the systolic blood pressure (103 ± 2.7, 86 ± 2.4 vs. 156 ± 7.83 mmHg, *p* < 0.05), intima-media thickness (101.28 ± 3.4, 93.91 ± 2.93 vs. 143.78 ± 3.31 µM), vasoconstriction percentage induced by phenylephrine (102.5%, 88.3%, vs. 51.8%), sICAM-1 (0.49, 0.26 vs. 0.56 pg/mL) and sVCAM-1 (0.39, 0.25 vs. 0.45 pg/mL). GP extract supplementation increased vasorelaxation percentage induced by acetylcholine (78.4% vs. 47.3%) and sodium nitroprusside (84.2% vs. 53.7%), increased changes in plasma nitric oxide level (1.25%, 1.31% vs. 1.9%), and suppressed the elevation of TNF-α (0.39 vs. 1.02 pg/mL), IL-6 (0.43 vs. 0.77 pg/mL) and CRP (0.29 vs. 0.69 ng/mL) in the PM groups.

**Conclusions:**

GP extract might improve vascular dysfunction by suppressing the inflammatory response, consequently preventing blood pressure elevation.

## Introduction

Menopausal women tend to develop cardiovascular diseases (Barrett-Connor 2013; Fairweather 2014) as in male individuals. Globally, the mortality rate of cardiovascular diseases is increasing, therefore prevention of cardiovascular diseases, particularly atherosclerosis, is important (Mosca et al. 2011; Yao et al. 2019). Atherosclerosis is a chronic inflammatory disease. It is indicated by the thickening and loss of elasticity of the arterial walls, leading to endothelial dysfunction and increasing blood pressure (Dinh et al. 2014). The endothelium acts as an interface between circulating blood or lymph in the lumen of the vessel wall, and protects the interior surface of blood and lymphatic vessels (Pfeiffer et al. 2008). An endothelium dysfunction occurs when the endothelium cannot maintain its vascular homeostasis due to the loss of balance between vasodilation and vasoconstriction (Rajendran et al. 2013).

Various inflammatory cells, specifically monocytes and macrophages, play a major role in the development, progression, and rupture of atherosclerotic plaques (Camici et al. 2012; Husain et al. 2015). The oxidation of low-density lipoprotein (LDL) triggers the release of chemokines that attract blood monocytes and T lymphocytes, activates endothelium and allows the adherence and entry of vascular cell adhesion molecule-1 (VCAM-1), intracellular cell adhesion molecule-1 (ICAM-1), E and P-selectin into the sub-endothelial space (Cook-Mills et al. 2011). Monocytes undergo differentiation into mature macrophages, engulf the excessive lipoproteins, and form cholesterol-enriched foam cells. The foam cells and lymphocytes release cytokines, such as interleukin-6 (IL-6), tumour necrosis factor-alpha (TNF-α) and monocyte chemotactic protein-1, and maintain a constant state of chronic vascular inflammation (Fardel 2013).

The production of uncontrolled reactive oxygen species (ROS) from endothelial nitric oxide synthase (eNOS) and nicotinamide-adenine dinucleotide phosphate (NADPH) oxidase reduces the bioavailability of nitric oxide (NO) (Vogiatzi et al. 2009) and enhances its inactivation, causing further impairment of the endothelium-dependent vasodilation. NO is synthesized primarily by eNOS within the blood vessel wall. Thus, the balance between NO production and ROS within the vascular wall determines NO accessibility to dilate blood vessels (He et al. 2019). eNOS may produce NO through its oxygenase function and superoxide reductase function. This applies to the ability of the blood vessels to maintain typical homeostasis and remain dilated. It also impacts the progression and development of atherosclerotic plaques with the tendency to rupture, and the precipitation of thrombosis. Prolonged use of statins to manage and control atherosclerosis might cause side effects to the liver enzymes due to the elevation of aminotransferase level (Kashani et al. 2006). The prolonged use of the drug may also cause damages to the skeletal muscles, for instance, such as myopathy (Zhang et al. 2014; Muntean et al. 2017).

*Gynura procumbens* (Lour.) Merr. (GP) (Asteraceae), commonly known as ‘Sambung nyawa’ in Malaysia, is used traditionally to treat various ailments, such as fever, rashes, constipation, high blood pressure, diabetes mellitus, hypertension, urinary tract infection, and kidney disease (Puangpronp et al. 2010; Mou and Dash 2016). Previous studies have revealed the active constituents of GP extract were made up of flavonoids, saponins, tannins, terpenoids and sterol glycosides (Kaewseejan et al. 2015; Ashraf 2019; Manogaran et al. 2019). Other studies reported that the leaf extract of GP contained several potential antioxidant components including kaempferol, astragalin, kaempferol-3-*O*-rutinoside, rutin, caffeic acid and chlorogenic acid (Rosidah et al. 2008; Ahmad Nazri et al. 2019).

Due to these beneficial chemical constituents, researchers have extensively investigated the phytoconstituents of GP to demonstrate their therapeutic benefits on modulating hypoglycaemic (Algariri et al. 2014; Sathiyaseelan et al. 2020), hypertensive and cardioprotective (Hoe et al. 2011; Poh et al. 2013), antioxidant (Krishnan et al. 2015; Ahmad Nazri et al. 2019), and inflammatory responses (Ning et al. 2019). Wong et al. (2015) showed that kaempferol, a major constituent of GP, inhibited the growth of *Plasmodium falciparum* 3D7 by modulating GSK3β (Ser9) enzyme, which was critical in regulating pathogen-induced inflammatory responses through phosphorylation in the life cycle of the plasmodial parasite during infection. Kim and Kim (2011) showed that astragalin (kaempferol-3-*O*-glucoside) inhibited lipopolysaccharide (LPS)-induced pro-inflammatory cytokine mediators, interleukin-6 (IL-6), IL-8, matrix metalloproteinase-1 (MMP-1), matrix metallopeptidase-9 (MMP-9), tumour necrosis factor-alpha (TNF-α), and interferon-gamma (IFN-γ) through nuclear factor kappa B (NF-κB) expression in LPS-activated mouse macrophages. Moreover, astragalin increased the expression of an anti-inflammatory cytokine, IL-10 when supplemented with GP extract (Wong et al. 2015). Ning et al. (2019) reported that 80% ethanol GP extract has high potential in preventing inflammation by regulating NO production and iNOS expression in RAW264.7 macrophages-LPS stimulated cells. This study showed that the pre-treatment with 250 μg/mL of GP could increase the cell viability by 90% and suppressed the expression of iNOS protein and NO production in LPS-stimulated RAW264.7 macrophages. In addition, astragalin might be the phytoconstituent of GP extract that exerted an anti-inflammatory effect in the LPS-stimulated RAW 264.7 cells.

Consequently, our study was carried out to investigate the anti-inflammatory effect of 80% ethanol GP extract on vascular dysfunction due to inflammation in postmenopausal rats fed with a high-fat diet. Despite many comprehensive studies on the anti-inflammatory mechanism, we believe the current study is the first to investigate the anti-inflammatory effect of GP extract as a prominent therapeutic drug candidate to treat illnesses related to inflammation in postmenopausal women.

## Material and methods

### Plant material and ethanol extracts of *G. procumbens*

A total of 50 kg fresh whole plant of GP was obtained from Semenyih (Brightmark Sdn. Bhd.), Selangor. The plant was identified and deposited at the Herbarium of Universiti Kebangsaan Malaysia (UKM), Bangi, Malaysia, with the voucher number of UKMB40375. The plant was cleaned and air-dried for two weeks. The dried plant material was ground into powder and subjected to extraction. The extraction of GP was carried out as previously reported by Ahmad Nazri et al. (2019).

Briefly, GP extraction was performed by macerating 1900 g of powdered dried plant material with 80% ethanol at a ratio of 1: 20 (w/v) for 24 h. The powdered dried plant was soaked in 80% ethanol for 3 days, followed by a continuous stirring for 3 times per day at room temperature. After three days, the solvents were filtered through Whatman No.1 filter paper (Whatman, England) and the filtrates were pooled. The excess solvent was evaporated by using a rotary evaporator at 55 °C to obtain 92 g of crude extract. The ethanol extract was kept in amber bottles at −4 °C to let the solvent dry, followed by freeze-drying before further use. The major compounds present in the extract have been identified previously by us, by using a validated HPLC method and LC-MS/MS analysis (Ahmad Nazri et al. 2019). The HPLC chromatogram and LC-MS/MS data are shown in Supplementary 1 (S1).

### Animals

Forty-eight healthy female Sprague-Dawley rats aged 3 months (weighing 250–300 g) were obtained from the Laboratory of Animal Resource Unit, UKM, Malaysia. They were placed in plastic cages, provided with regular bedding, and kept at room temperature of 27 ± 2 °C, 12 h light/dark cycle accompanied with adequate ventilation. The animals were supplied with commercial pellet food (Gold Coin, Selangor, Malaysia) and water, *ad libitum* access. The food taken was weighed weekly at an average of 50 g/day/rat. Animal handling procedures in this study were approved by the Animal Ethical Committee of UKM (UKMAEC) with the approval number BIOK/PP/2016/ZAKIAH/28-SEPT./779-SEPT.-2016- JAN.-2019.

### Experimental protocol

The rats were acclimatized for two weeks before being subjected to any experimental procedures to avoid stress. Forty-eight rats were randomly divided either into the postmenopausal (PM) (ovariectomized; *n* = 24) groups to produce a postmenopausal oestrogen-deficient state or the sham-operated (non-ovariectomized; *n* = 24) group to simulate surgical stress. Ovariectomized procedures were conducted with aseptic techniques established by Khajuria et al. (2012).

Briefly, the rats were fasted for 2–3 h without restricting water intake. They were anaesthetized by intraperitoneal injection (ketamine at 80 mg/kg and xylazine at 10 mg/kg) based on 0.1 mL per 100 g of body weight. The rats were examined on several parameters to avoid excessive depression of cardiac and respiratory functions or insufficient anaesthesia. The body temperature of the rats was also monitored using a rectal thermometer. Physiological stress experienced by the animal could induce hypothermia and would prolong the recovery period, potentially causing fatality. Then, the ventral part of the abdominal region was shaved and swabbed with ethanol. A transverse peritoneal incision of approximately 1 cm was made through the abdominal skin with a surgical blade. The fallopian tubes were clamped to prevent over-bleeding before both ovaries were identified and removed. A sterile braided suture was performed around the sectioned area in two layers with 4/0 absorbable catgut suture (inner skin) and 4/0 nonabsorbable silk suture (outer skin). Iodine was sprayed on the stitched area followed by post-operative antibiotic (Baytril^®^ 5%) intramuscular injection for one week to prevent infection. Similar procedures were applied to sham animals without removing the ovaries. All rats were left to recuperate for one week and then re-grouped into their home cages before commencing the treatment. The success of the ovariectomy procedure was verified by oestrous cycle assessment, by examining and evaluating the appearance of the vulva at different stages of oestrous cycle (Ajayi and Akhigbe 2020).

Animals were randomly divided into eight groups comprising of six animals per group: sham rats; (i) control, (ii) 250 mg/kg GP, (iii) 500 mg/kg GP, (iv) atorvastatin (AVT); and postmenopausal rats; (v) PM, (vi) PM + 250 mg/kg GP, (vii) PM + 500 mg/kg GP, (viii) PM + ATV groups. Each group received either 250 or 500 mg/kg of GP or ATV at 10 mg/kg by oral gavage for 6 months concurrently with or without 2% cholesterol chow mixed with 15% (w/w) of five-times heated palm oil (5HPO). GP or ATV was administered in a maximum volume of 1 mL to each rat accordingly (Ahmad Nazri et al. 2019). Toxicity effects from GP and ATV supplementation were verified by liver function test. During the treatment, the systolic blood pressure (SBP) was monitored, and blood was collected at the interval of three months. At the end of the treatment period (6 months), the rats were fasted overnight before being sacrificed by exposing them to a low concentration of diethyl ether from a vaporizer (Yadav and Sisodia 2020). Inhalation of low concentration of diethyl ether for at least 2 min could lessen the irritation to the rats (Aguwa et al. 2020).

### Preparation of five-time heated palm oil

The high-fat diet used in this study was prepared as described previously (Owu et al. 1998). The diet was formulated and prepared by mixing 15% (w/w) of prepared 5HPO and 2% cholesterol ground chow pellet. The palm oil used was supplied by a local manufacturer (Cap Buruh, Lam Soon Edible Oil, Kuala Lumpur) and 2% cholesterol chow pellet was from Singapore (MP Biomedicals). Approximately 1 kg of sweet potatoes was cleaned, peeled, and sliced before deep-fried in pre-heated (180 °C) 2.5 L palm oil in a stainless-steel wok. Sliced sweet potatoes were deep-fried for 15 min and discarded before continuing to deep-fry new batches of sweet potatoes. The remaining heated oil was left to cool for 5 h without adding fresh oil for the subsequent frying process. These procedures were repeated four times to obtain 5HPO. Subsequently, a 2% cholesterol grounded chow pellet diet was mixed with 15% (w/w) of the prepared 5HPO. The mixture was moulded into ‘ball-like’ shapes and dried in an oven at 70 °C overnight.

### Collection of blood and aorta tissue

Whole blood samples were drawn at months 0, 3, and 6 via an orbital sinus. The rats were put in an unconscious state by short exposure to a low concentration of diethyl ether from a vaporizer. A volume of 6 mL whole blood was collected using a capillary tube inserted into the heparin blood tubes, immediately centrifuged for plasma at 1000 × *g* for 10 min, and kept on ice promptly. The plasma was separated, aliquoted, and stored at −80 °C for biochemical analysis. The thoracic aorta tissues were excised out, cleaned, and cut into few segments immediately after sacrificing the rats for vascular reactivity. Some of them were fixed in 10% formalin for one week for histomorphometry study and aortic endothelial expression analysis.

### Measurement of systolic blood pressure

The systolic blood pressure (SBP) was measured using a non-invasive tail-cuff method (CODA Powerlab, AD Instruments, NSW, Australia) at the interval of 3 months treatment period for 6 months (Adam et al. 2009). This system uses volume pressure recording (VPR) by considering the changes of volumes occurring in the tail to measure the SBP. Briefly, the rats were pre- warmed under sunlight for 10 min to adapt to the environment before measuring the SBP. Concurrently, a strainer tube was swabbed with 70% ethanol, and the CODA system was calibrated according to the standard settings recommended by the manufacturer. A rat was placed in the strainer tube where its tail was adjusted in a comfortable position to prevent excessive movement of the occlusion cuff near the proximal end of the strainer tube. Five valid readings of SBP were taken, and the mean of SBP was calculated for each rat.

### Aortic histomorphometry analysis

The isolated aortal tissue was stored in 10% formalin and dried at room temperature. Then, the tissue was placed in the paraffin block and embedded with paraffin wax. The paraffin wax block was excised into 5 µm thin sections using a rotary microtome (LEICA RM2235, Walldorf, Germany), and stained with Verhoeff-van Gieson (VvG) dye solution to identify the elastic fibres and smooth muscle cells. Next, the slides were viewed, and the images were acquired using a contrast phase microscope (BX50; Olympus) by photographing four regions (0°, 90°, 180°, and 270°) of each slide, including the tunica intima (TI), tunica media (TM), and intima-media thickness (Baruscotti et al. 2010). The average thickness for each region was analysed with the Axio Vision LE software and calculated following the equations below. This method was conducted according to a previous method by Fernandes-Santos et al. (2009).

$$\mathrm{Average}\;\mathrm{thickness}\;of\;TI\;\mathrm{and}\;TM\;(\mu m)=\frac{\mathrm{Measurements}\;at\;0{}^{\circ},\;90{}^{\circ},\;180{}^{\circ},\;270{}^{\circ}}{4}$$

IMT (μm)=Average thickness of TI+ Average thickness of TM

### Measurement of vascular reactivity

Vascular reactivity was determined using the descending thoracic aorta (Ajay and Mustafa 2006). The aorta was dissected while the excess fat and the connective tissues, were removed and cut into 3 mm width ring segments. The aortic ring segments were immersed in 25 mL freshly prepared filtered Krebs salts solution or the bathing solution. A mixture of 95% oxygen and 5% carbon monoxide were gassed continuously into the bathing solution, and the temperature was maintained at 37 °C. A force-displacement transducer (FT03E, Grass Instruments West Warwick, RI, USA) was pre-set to a recording system (MacLab Model 8 S, AD Instruments, Castle Hill, NSW, Australia) to measure the isometric tension (g) of the aortic rings.

At the start of the experiment, the equilibration process was performed by immersing the aortic ring segments in the bathing solution for 30 min until the basal tension readings were stable and appeared as 1 g. Then, the high content of K + traces was washed out. Subsequently, an isotonic KCl solution (high K+, 80 mM) was added intermittently into the bathing solution until a maximal tension was achieved. In order to test the relaxation responses to Ach (10–10 to 10–6 M) and SNP (10–10 to 10–6 M), the aortic rings were pre-contracted with phenylephrine (PE; 10–10 to 10–6 M). These steps were repeated on other freshly isolated aortic rings. Only the endothelial intact rings with more than 50% relaxation to Ach were used. Relaxation responses to cumulative concentrations of Ach and SNP were calculated as percentage inhibition of PE- induced maximal contraction, and a dose-response curve was drawn using the nonlinear regression (Prism version 2.0, Graphpad Software, USA). Relaxation responses to Ach or SNP were plotted as the percentage relaxation versus log concentration of Ach or SNP. The pEC50 (–log of median effective concentration) and percentage relaxations at the highest concentration (Rmax) were calculated from the graph (Machha and Mustafa 2005; Muharis et al. 2010; Suhaimi et al. 2020).

### Measurement of nitric oxide

Nitrite metabolite was measured to obtain nitric oxide content following a previous method by Nurul-Iman et al. (2013). Briefly, blood separation was conducted by centrifugation at 1500 × *g* for 10 min to obtain the plasma, and it was stored at −80 °C. Approximately 50 µL of plasma sample was pipetted into a 96-well plate with 50 µL of pre-filled modified Griess reagent (Sigma-Aldrich, St. Louis, MO, USA). The 96-well plate was covered with aluminium foil and incubated for 15 min at room temperature. The absorbance reading of nitrite concentration was measured at 540 nm using the EnSpire Multimode Plate Reader (Perkin Elmer, USA).

### Measurement of inflammatory biomarkers

The level of inflammatory biomarkers, such as IL-6 (E-EL-R0015), TNF-α (E-EL-R1037), and CRP (E-EL-R0022) were measured using Elabscience Biotechnology (USA) ELISA kit in a 96-well plate. All reagents, samples, and standards were prepared according to the manual provided with the kit. Briefly, the blood was collected into heparin tubes and separated for 15 min at 1000 × *g* at 4 °C to obtain the plasma. Then, the plasma was collected and added into the 96-well plate before incubating for 90 min at 37 °C. The absorbance of IL-6, TNF-α, and CRP was taken at 450 nm using the EnSpire Multimode Plate Reader (Perkin Elmer, USA) and compared with the standard curve generated.

### Measurement of aortic endothelial expression of sCAM-1 and sVCAM-1

The endothelial expression in aortic tissues, including sICAM-1 (E-EL-R0046) and sVCAM- 1 (E-EL-R1061), was measured using the ELISA kit (Elabscience Biotechnology, USA). Tissue homogenates were prepared as recommended in the manual provided by the manufacturer. Briefly, minced aortic tissues were rinsed with ice-cold PBS (0.01 M, pH = 7.4) to wash out traces of blood. The tissues were homogenized in PBS with a glass homogenizer in a ratio of 1 g of tissue in 9 mL of PBS. The tissue homogenates were subjected to sonication using an ultrasonic cell disrupter. Subsequently, the homogenates were centrifuged for supernatant at 5000 × *g* for 5 min. Absorbance reading of the supernatants was measured using the EnSpire Multimode Plate Reader (Perkin Elmer, USA) at 450 nm absorbance. The absorbance values were obtained and calculated by the software provided and compared with the generated standard curve.

### Liver function test

Biochemical analysis was carried out to determine the serum concentration of total protein, albumin, globulin and the activities of liver enzymes such as alkaline phosphatase (ALP), aspartate aminotransferase (AST) and alanine aminotransferase (ALT) using diagnostic kits (Roche, NJ, USA).

### Statistical analysis

The data were expressed as mean ± standard deviation (SD). Normality of the data was determined by the Kolmogorov-Smirnov test. Statistical differences were determined using a two-way analysis of variance (ANOVA), followed by Tukey HSD *post* *hoc* test. A value of *p* < 0.05 was considered to be significant. All data were analysed using SPSS for Windows version 20.0.

## Results

### *G. procumbens* extract supplementation reduced the systolic blood pressure in the postmenopausal rat

**Figure 1.**
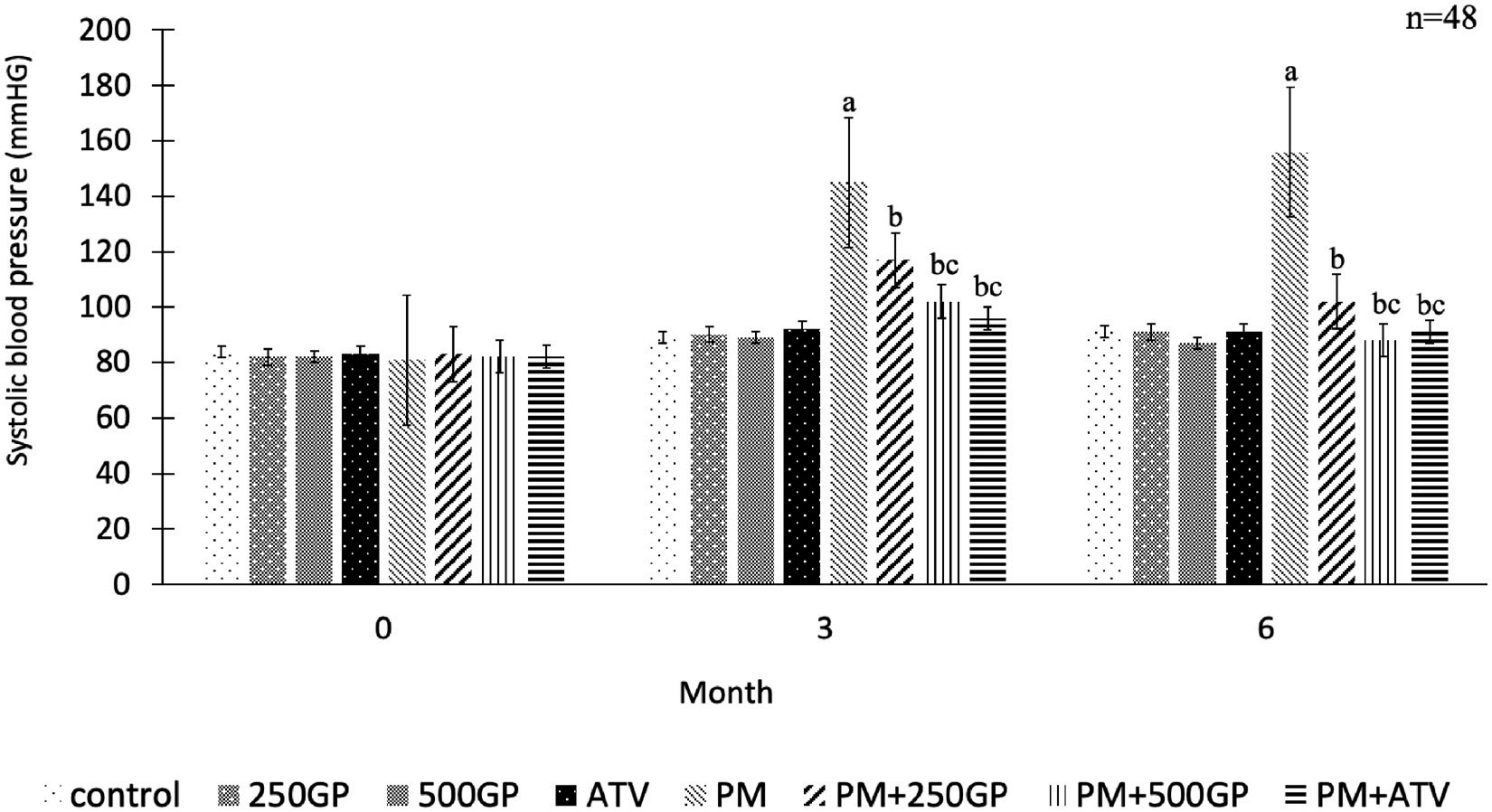
Effects of *Gynura procumbens* extracts on systolic blood pressure of sham and postmenopausal (PM) groups at 0, 3 and 6 months of supplementation. Data are means ± SEM. aIndicates a significant difference compared to control (*p* < 0.05), bIndicates a significant difference compared to PM group (*p* < 0.05) and c indicates a significant difference compared PM + 250GP (*p* < 0.05).

Systolic blood pressure in the PM groups increased significantly (*p* < 0.05) compared to the control group (Figure 1). The GP extract at 250 and 500 mg/kg reduced systolic blood pressure significantly (*p <* 0.05) in PM + 250GP and PM + 500GP groups compared to the PM group starting at month 3 until the end of the experiment (119 ± 3.7 mmHg to 103 ± 2.7 mmHg, 101 ± 4.2 mmHg to 86 ± 2.4 mmHg vs. 143 ± 7.81 to 156 ± 7.83 mmHg). A similar effect was presented by atorvastatin at month 3 and 6 in the PM + ATV group compared to the PM + 250GP group (98 ± 1.85 mmHg vs. 119 ± 3.7 mmHg to 95 ± 1.89 vs. 103 ± 2.7 mmHg) but no significant difference compared to PM + 500GP.

### *G. procumbens* extract supplementation reversed the effects of 5HPO in aortic histomorphometry of postmenopausal rat

**Figure 2.**
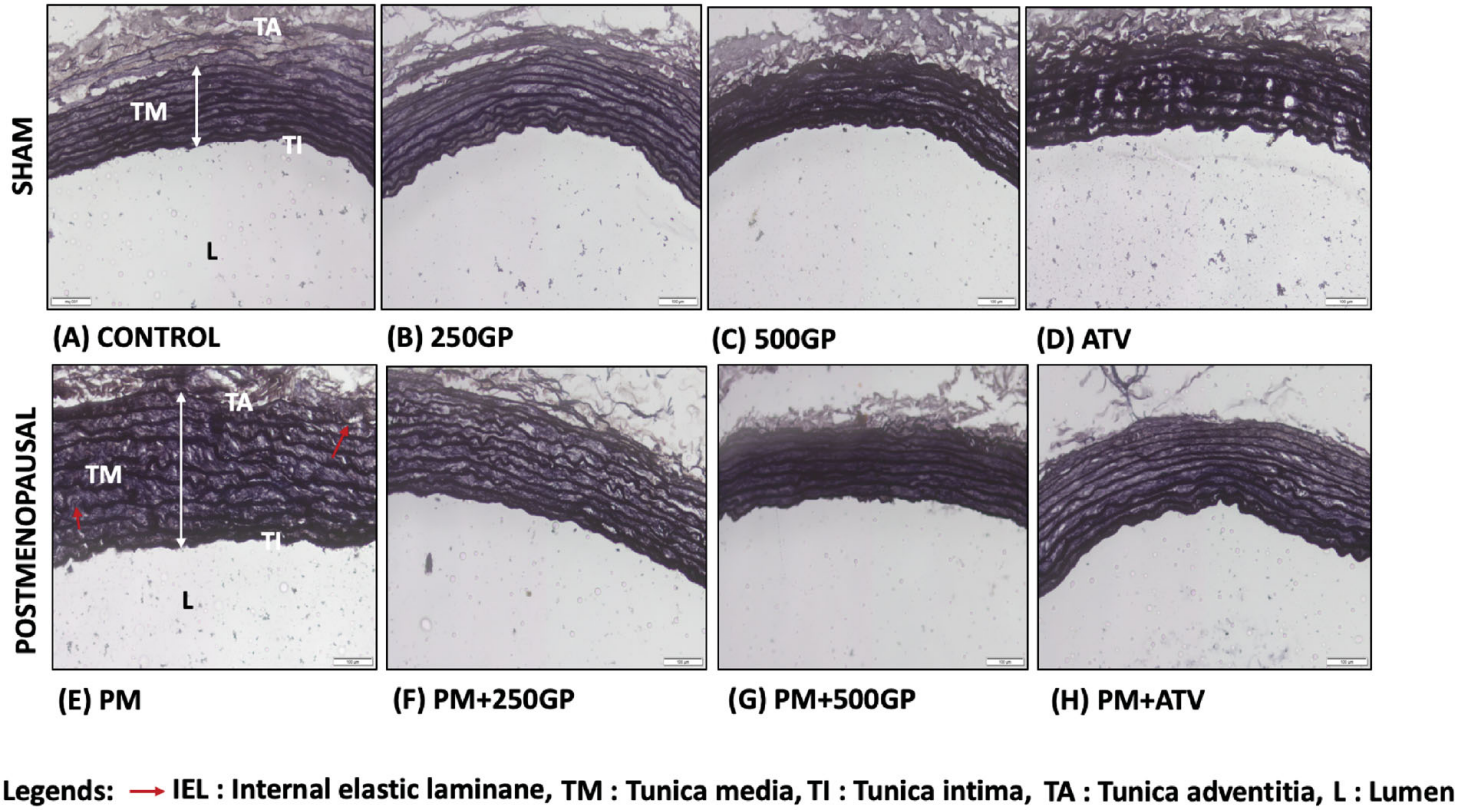
Photomicrograph showing the section of aortic tissues in (A) Control; (B) 250GP; (C) 500GP; (D) ATV; (E) postmenopausal (PM); (F) PM + 250GP; (G) PM + 500GP and (H) PM + ATV after 6 months of supplementation where TI: tunica intima, TM: tunica media, TA: tunica adventitia and L: lumen under Verhoeff-Van Gieson stain. (LM × 40).

The histology of aortic features in the sham (photo A–D) and postmenopausal (photo E–H) groups are illustrated in Figure 2. The endothelial cells (EC) nuclei were stained dark in the the control group, indicating a tunica intima (TI). Meanwhile, the EC appeared flattened, laid near to the internal elastic laminae (IEL). The IEL were depicted as continuous, thick, and wavy, as indicated by the white arrow in photo A. Meanwhile, the EC of the PM group was shown as a thick and dark nuclei layer along the vessel luminal surface (photo E). The IEL appeared disrupted and discontinuous. However, supplementation with GP in the PM group (photo F and G) reversed the effect by decreasing the thickening of tunica media (TM). However, in the present study, the thickness of tunica intima (TI) did not significantly show any significant changes compared to the control group. Table 1 shows the measured aortic thickness of TI, TM, and the ratio of IMT. No significant changes were observed in the TI thickness of the PM group with GP supplementation compared to the control group. The thickness of TM significantly increased (143.8 ± 2.65 µM) in the PM group compared to the control (50.03 ± 2.52 µM). Similarly, intima-media thickness also increased in the PM group. Supplementation with GP at 250 and 500 mg/kg of body weight significantly decreased the thickness of TM and IMT compared to the PM group. Atorvastatin also increased IMT in the PM group.

**Table 1.** Aortic morphometric measurements of tunica intima (TI), tunica media (TM) and intima-media thickness (IMT) in sham and postmenopausal rats.

Group	TI (µM)	TM (µM)	IMT (µM)
Control	3.22 ± 0.19	50.03 ± 2.52	53.25 ± 3.2
250GP	3.83 ± 0.29	54.4 ± 3.10	58.23 ± 3.5
500GP	3.56 ± 0.27	51.7 ± 1.99	55.26 ± 2.31
ATV	3.37 ± 0.24	57.3 ± 5.07	60.67 ± 5.2
PM	4.98 ± 0.39	**^a^**143.8 ± 2.65	**^a^**148.78 ± 3.31
PM + 250GP	4.08 ± 0.31	**^b^**97.2 ± 3.44	**^b^**101.28 ± 3.4
PM + 500GP	4.11 ± 0.32	**^b^**89.8 ± 2.84	**^bc^**93.91 ± 2.93
PM + ATV	4.78 ± 0.35	101.7 ± 2.21	**^c^**106.48 ± 2.71

Data are expressed as mean ± standard deviation. ^a^Indicates a significant difference compared to control (*p* < 0.05), ^b^Indicates a significant difference compared to PM (*p* < 0.05) and ^c^Indicates a significant difference compared to PM + 250GP.

### *G. procumbens* extract supplementation improved the aorta vasorelaxation and vasoconstriction response in postmenopausal rat

**Figure 3.**
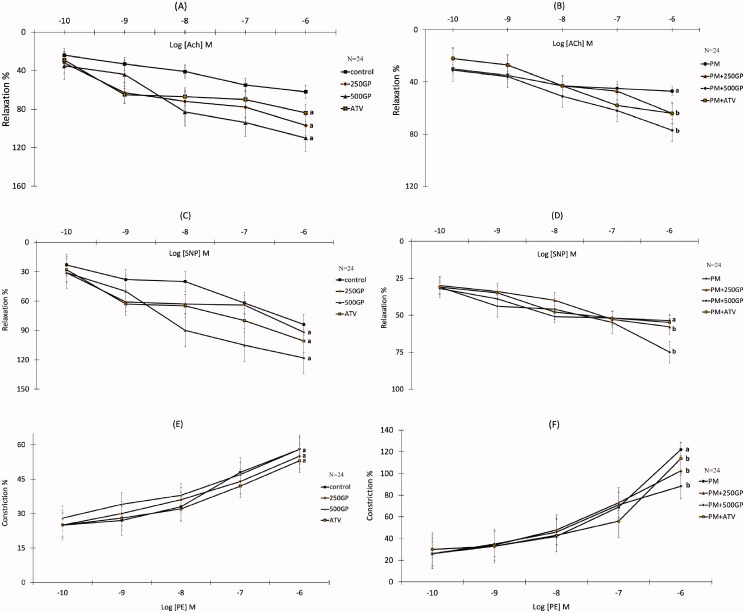
(A) Effects of *Gynura procumbens* extracts on aorta relaxation response induced by Ach in sham groups after 6 months of supplementation. Data are means ± SEM. aIndicates a significant difference compared to control (*p* < 0.05) and bIndicates a significant difference compared to PM group (*p* < 0.05). **(B)** Effects of *Gynura procumbens* extracts on aorta relaxation response induced by Ach in postmenopausal groups after 6 months of supplementation. Data are means ± SEM. aIndicates a significant difference compared to control (*p* < 0.05) and bIndicates a significant difference compared to PM group (*p* < 0.05). **(**C) Effects of *Gynura procumbens* extracts on aorta relaxation response induced by SNP in sham groups after 6 months of supplementation. Data are means ± SEM. aIndicates a significant difference compared to control (*p* < 0.05) and bIndicates a significant difference compared to PM group (*p* < 0.05). **(**D) Effects of *Gynura procumbens* extracts on aorta relaxation response induced by SNP in postmenopausal groups after 6 months of supplementation. Data are means ± SEM. aIndicates a significant difference compared to control (*p* < 0.05) and bIndicates a significant difference compared to PM group (*p* < 0.05). **(E)** Effects of *Gynura procumbens* extracts on aorta constriction response induced by PE in sham groups after 6 months of supplementation. Data are means ± SEM. aIndicates a significant difference compared to control (*p* < 0.05) and bIndicates a significant difference compared to PM group (*p* < 0.05). **(** F) Effects of *Gynura procumbens* extracts on aorta constriction response induced by PE in postmenopausal groups after 6 months of supplementation. Data are means ± SEM. aIndicates a significant difference compared to control (*p* < 0.05) and bIndicates a significant difference compared to PM group (*p* < 0.05).

The presence of the highest concentration of acetylcholine (Ach, 10–6 M; Figure 3(A)) and sodium nitroprusside (SNP, 10–6 M; Figure 3(C)) reduced (*p <* 0.05) the relaxation response by 47.3% and 53.7% in the PM group compared to the control group, respectively. Conversely, 250 and 500 mg/kg of GP supplementation significantly increased (*p <* 0.05) the relaxation responses to Ach (Figure 3(B)) and SNP (Figure 3(D)) in the PM group compared to the untreated PM group. However, no significant difference in relaxation response to Ach and SNP was shown in the PM of the ATV-treated group compared to the PM group. The contractile response of the thoracic aorta was induced by phenylephrine (PE, 10–6 M). Vasoconstriction of aortic rings in response to PE was significantly augmented in the PM group compared to the control group (Figure 3(E)). Supplementation with GP at 250 and 500 mg/kg, and 10 mg/kg of ATV significantly reversed (*p* < 0.05) the vasoconstriction responses by 102.5%, 88.3%, and 113.7%, respectively, in the PM aortic rings (Figure 3(F)).

### *G. procumbens* extract supplementation increased plasma nitric oxide level in postmenopausal rat

**Figure 4.**
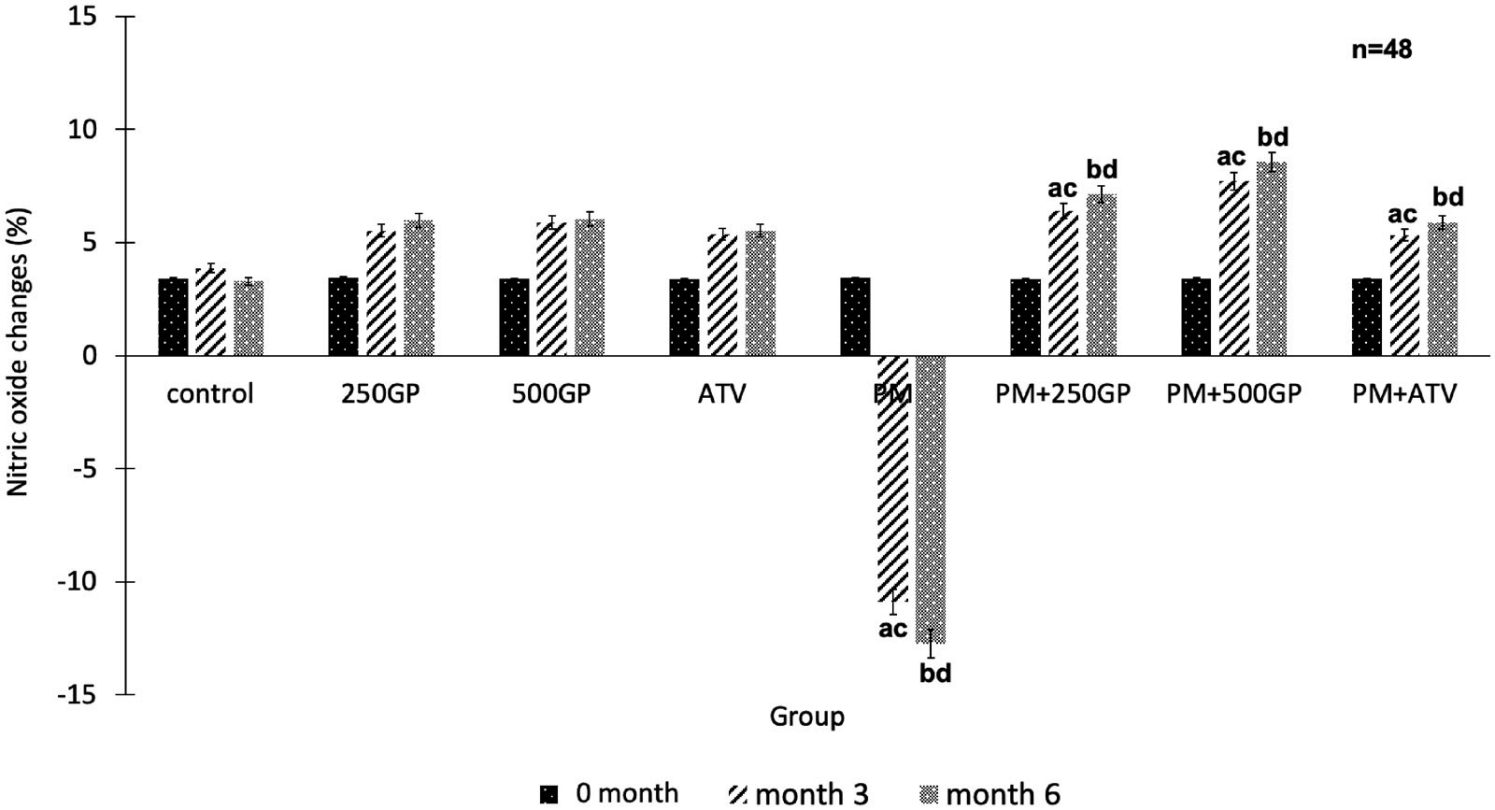
Effects of *Gynura procumbens* extracts on nitric oxide level in sham and postmenopausal (PM) groups at 0, 3 and 6 months of supplementation. Data are means ± SEM. aIndicates a significant difference compared to control in month 3 (*p* < 0.05) and bIndicates a significant difference compared to control in month 6 (*p* < 0.05), cIndicates a significant difference compared to PM in month 3 (*p* < 0.05) and dIndicates a significant difference compared to PM in month 6 (*p* < 0.05).

Figure 4 shows that the plasma NO level decreased significantly (*p <* 0.05) in the PM group compared to the control at month 3 (–10.9 ± 1.7% vs. 3.86 ± 1.4%) and 6 (–12.74 ± 1.9% vs. 3.29 ± 0.6%). Supplementation with GP at 250 mg/kg (6.4 ± 0.3%, 7.14 ± 0.7%) and 500 mg/kg (7.72 ± 1.25%, 8.56 ± 1.31%) to the PM group increased the plasma NO level starting at month 3 until the end of the experiment compared to the untreated PM group. Administration of atorvastatin (4.89 ± 1.3% and 5.21 ± 1.5%) also showed a similar effect by increasing the plasma NO level in the PM group.

### *G. procumbens* extract supplementation reduced inflammatory biomarkers in postmenopausal rat

**Figure 5.**
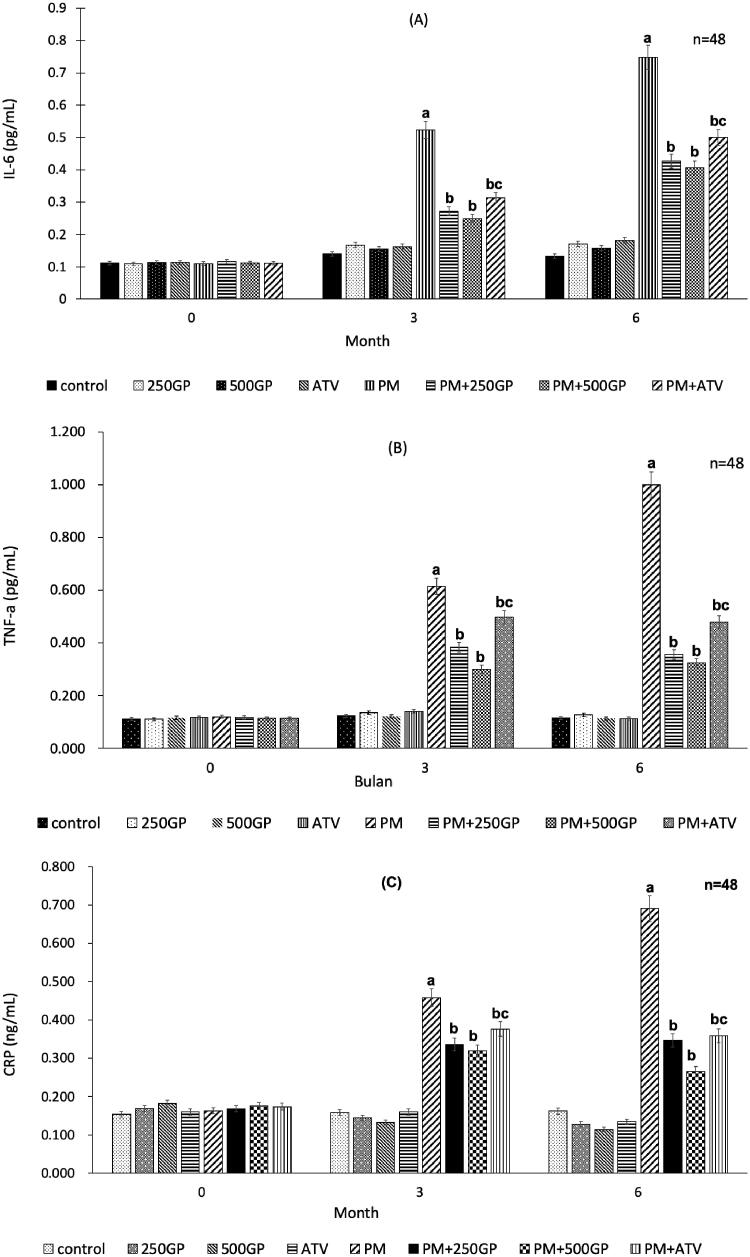
(A) Effects of *Gynura procumbens* extracts on IL-6 level in sham and postmenopausal groups at 0, 3 and 6 months of supplementation. Data are means ± SEM. aIndicates a significant difference compared to control (*p* < 0.05) and bIndicates a significant difference compared to PM group (*p* < 0.05), cIndicates a significant difference compared to PM + 250GP (*p* < 0.05). **(B)** Effects of *Gynura procumbens* extracts on TNF-alpha level in sham and postmenopausal groups at 0, 3 and 6 months of supplementation. Data are means ± SEM. aIndicates a significant difference compared to control (*p* < 0.05) and bIndicates a significant difference compared to PM group (*p* < 0.05), cIndicates a significant difference compared to PM + 250GP (*p* < 0.05). **(**C) Effects of *Gynura procumbens* extracts on CRP level in sham and postmenopausal groups at 0, 3 and 6 months of supplementation. Data are means ± SEM. aIndicates a significant difference compared to control (*p* < 0.05) and bIndicates a significant difference compared to PM group (*p* < 0.05), cIndicates a significant difference compared to PM + 250GP (*p* < 0.05).

Plasma inflammatory biomarkers level, IL-6 (Figure 5(A)), TNF-α (Figure 5(B)) and CRP (Figure 5(C)) increased significantly (*p <* 0.05) in the PM group after 3 months being fed with the high-fat diet compared to the control group. The reduction of inflammatory biomarkers level was observed with GP supplementation at 250 mg/kg (at month 3: IL-6, 0.28 pg/mL vs. 0.55 pg/mL; TNF- α, 0.38 pg/mL vs. 0.63 pg/mL; CRP, 0.33 ng/mL vs. 0.47 ng/mL; and at month 6: IL-6, 0.42 pg/mL vs. 0.77 pg/mL; TNF- α, 0.35 pg/mL vs. 1.02 pg/mL; CRP, 0.35 ng/mL vs. 0.69 ng/mL) and 500 mg/kg (at month 3: IL-6, 0.27 pg/mL vs. 0.55 pg/mL; TNF- α, 0.37 pg/mL vs. 0.63 pg/mL; CRP, 0.34 ng/mL vs. 0.47 ng/mL; and at month 6: IL-6, 0.43 pg/mL vs. 0.77 pg/mL; TNF- α, 0.39 pg/mL vs. 1.02 pg/mL; CRP, 0.29 ng/mL vs. 0.69 ng/mL). A similar effect was observed with atorvastatin treatment in the PM + ATV group.

### *G. procumbens* extract supplementation reduced adhesion molecules in postmenopausal rat

**Figure 6.**
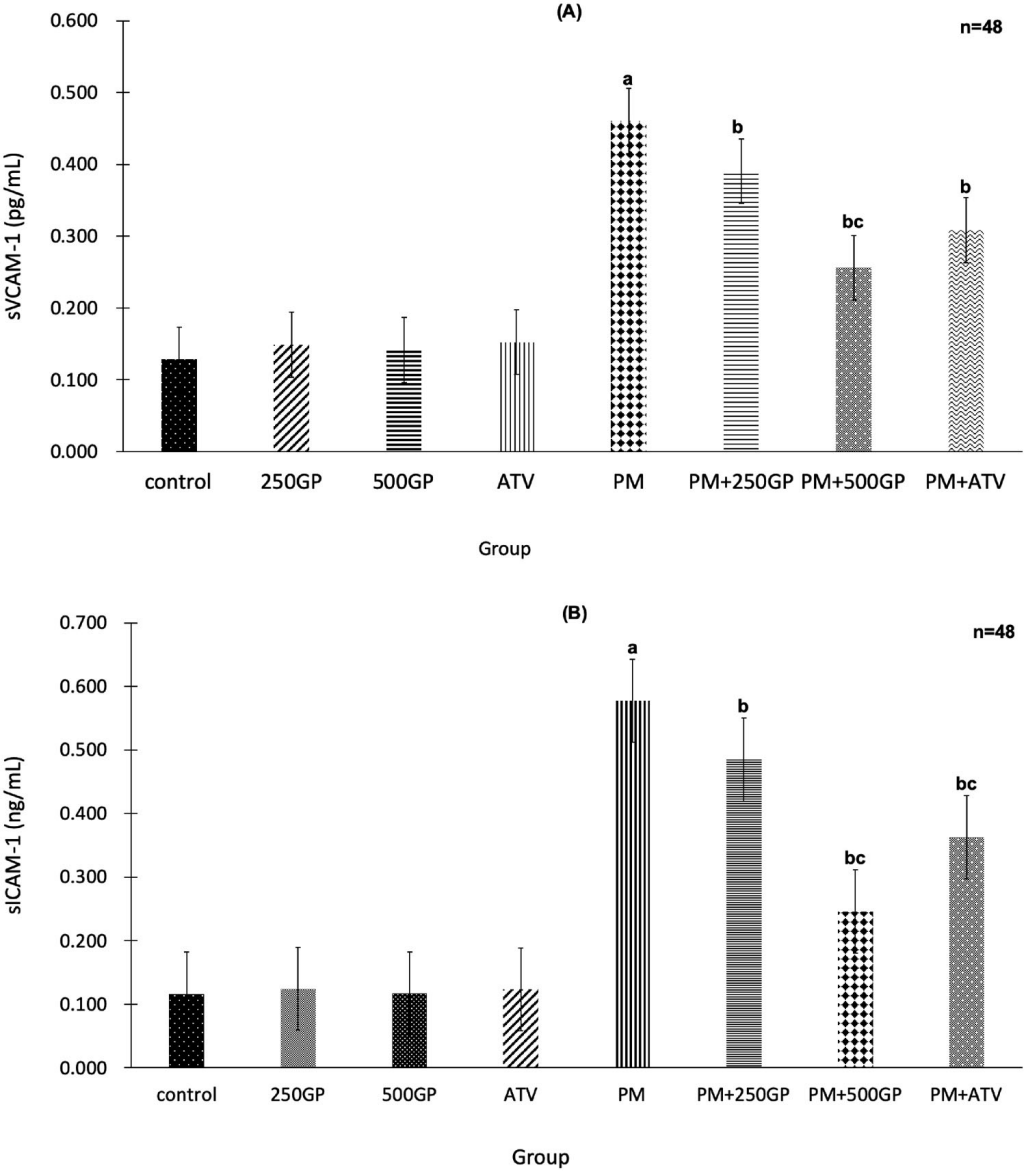
(A) Effects of *Gynura procumbens* extracts on sVCAM-1 level in sham and postmenopausal groups at 6 months of supplementation. Data are means ± SEM. aIndicates a significant difference compared to control (*p* < 0.05) and bIndicates a significant difference compared to PM group (*p* < 0.05), cIndicates a significant difference compared to PM + 250GP (*p* < 0.05). **(B)** Effects of *Gynura procumbens* extracts on sICAM-1 level in sham and postmenopausal groups at 6 months of supplementation. Data are means ± SEM. aIndicates a significant difference compared to control (*p* < 0.05) and bIndicates a significant difference compared to PM group (*p* < 0.05), cIndicates a significant difference compared to PM + 250GP (*p* < 0.05).

The significant (*p <* 0.05) increase of sVCAM-1 and sICAM-1 expression in the PM group compared to the control group is shown in Figure 6(A and B), respectively. GP extract supplementation at 250 and 500 mg/kg reduced the expression of sVCAM-1 and sICAM-1 in PM + 250GP and PM + 500GP groups compared to the PM group (sVCAM-1: 0.39 pg/mL, 0.25 pg/mL vs. 0.45 pg/mL; and sICAM-1: 0.49 pg/mL, 0.26 pg/mL vs. 0.56 pg/mL). Atorvastatin also reduced sVCAM-1 and sICAM-1 expression in the PM + ATV group compared to the untreated PM group.

### Liver function test

Serum level concentration of total proteins, albumin, globulin, albumin/globulin ratio and the activities of liver enzymes (ALP, AST and ALT) of the PM group and PM treated group did not differ from the control group (Table 2).

**Table 2.** Effects of *Gynura procumbens* extract and atorvastation (ATV) supplementation on liver functions test in sham and postmenopausal (PM) groups at 0 and 6 months of study.

Biochemical parameters	Reference range (Suckow et al. 2006)	Groups															
		Control		250GP		500GP		ATV		PM		PM + 250GP		PM + 500GP		PM + ATV	
		0	6	0	6	0	6	0	6	0	6	0	6	0	6	0	6
Total protein	45–84 (g/L)	46	63	44	62	45	57	45	66	45	89	44	72	44	65	45	78
Albumin	40–48 (g/L)	43	44	43	47	43	46	43	44	43	51	43	40	44	40	44	43
Globulin	12–20 (g/dL)	12	15	12	16	12	15	12	17	12	27	12	17	12	14	12	15
Bilirubin	1.2–2.0 (mg/dL)	1.3	1.4	1.3	1.5	1.2	1.4	1.2	1.5	1.3	2.4	1.2	1.6	1.2	1.3	1.2	1.4
Alkaline phosphatase (ALP)	132–312 (U/L)	134	133	133	143	134	139	133	200	133	314	133	204	133	200	133	205
Aspartate aminotransferase (AST)	77–157 (U/L)	78	88	74	79	78	80	78	81	78	163	78	127	79	121	78	130
Alanine aminotransferase (ALT)	22–224 (U/L)	95	127	99	120	94	115	95	124	125	236	96	205	94	199	95	211

## Discussion

Oestrogen exerts protective effects on the cardiovascular system among premenopausal women. During menopause, oestrogen and its protective effects are lost due to cardiovascular system deterioration in females, showing similar or higher intensity than age-matched males (Yang and Reckelhoff 2011). In this study, we used postmenopausal rats fed with a high-fat diet as a model to study the effects of GP extract on vascular dysfunction and inflammation. The high-fat diet used in this study consisted of 2% cholesterol diet, enriched with five-times heated palm oil (5HPO) to accelerate the hyperlipidemic state until the stage of the early event of atherosclerosis, following the same model as in the study by Ahmad Nazri et al. (2019).

They reported that GP supplementation reduced oxidative stress and prevented membrane cell damage by modifying through the modification of antioxidant enzyme activity and the lipid profile changes. These findings proposed that the major phenolic contents, chlorogenic acid, gallic acid, kaempferol, quercetin, and rutin, identified in GP extract were potent antioxidants and scavengers of ROS. Furthermore, the level of total cholesterol (TC), total triglycerides (TG), low-density lipoprotein (LDL) (Supplementary 2; S2) and malondialdehyde (MDA) were reduced in the postmenopausal group with GP supplementation, and they increased the high-density lipoprotein (HDL) level. The generation of reactive oxygen species (ROS) from the multiple-time heated oil caused the oil to undergo thermo-oxidation and altered the composition of fatty acids (Foudjo et al. 2019). This explained the damage occurred in vascular cells, impairment of endothelial vasodilation, and increased blood pressure (Mudau et al. 2012).

This study showed that systolic blood pressure (SBP) in the postmenopausal (PM) group increased following consumption of high-fat diet most probably due to the presence of peroxide radicals, and other ROS generated in the multiple-heated oil in the diet. A similar finding was reported previously, proving that multiple-heated oil in the diet elevated lipid peroxidation exceeding the maximum limit of peroxide value (10 mEq/kg) in foods (Ng et al. 2012; Suhaimi et al. 2020). However, the supplementation with GP extract at the dose of 250 and 500 mg/kg managed to reduce blood pressure to the normal level, and the dose of 500 mg/kg GP showed the potential effect as the ATV, the positive control.

This observation could be due to the lowering effect exerted by the GP extract on the endothelial cell function as determined by the total peripheral resistance (TPR) of the blood vessels (Leong et al. 2009). In addition to that, the putative bioactive compounds in the GP extract, probably the flavonoids, lower the blood pressure by inhibiting the angiotensin I- converting enzyme (ACE) activity, blocking the calcium channels of peripheral blood vessels, and subsequently preventing the increase in blood pressure (Poh et al. 2013). Moreover, we proposed that GP in the presence of phenolic compounds could retard lipid peroxidation by inhibiting fat absorption and impeding cholesterol metabolism in the liver (Shimoda et al. 2006).

### *G. procumbens* maintains the vascular endothelial structure and function

The consumption of a diet that contains heated oil disrupts the organization of the elastic lamellae, leading to increased intimal media (IM) thickness, and the development of atherosclerosis (Siti et al. 2017). Inflammation starts within the tunica adventitia (TA) rather than tunica intima (TI), and spreads inwards into the vasculature (Maiellaro and Taylor 2007). The observation on the Verhoeff-van Gieson (VvG) staining showed an internal elastic laminae (IEL) disturbance and thickening of the tunica media (TM), affirming the presence of aortic damage in the postmenopausal group. Significant disorder in TM and intima-media thickness (Baruscotti et al. 2010) indicates the presence of subclinical atherosclerosis (Targher et al. 2006). However, the absence of a significant difference in TI thickness might be due to the less effects on the foam cell formation in the tunica intima layer of the aortic wall than the cholesterol-induced atherosclerosis in the tunica media (Soufy et al. 2012). Our study was contradictory to another study that reported tunica intima thickness increased due to the appearance of the foam cell (Castro et al. 2009). However, the GP extract retained the typical histology and ultrastructure of the aorta, which was comparable to the controls from the sham group. The mechanism of GP extract action is most likely due to the presence of flavonoids, such as rutin, kaempferol, and quercetin, that have inhibitory impacts on the proinflammatory cytokines, such as TNF-α, IL-6, and NO (Saraphanchotiwitthayaa and Sripalakitb 2015). According to the current findings, GP exhibited a protective role in atherosclerotic lesion to maintain the aortic tissue integrity by reducing the degenerative changes in the intima area.

### *G. procumbens* improves endothelial function by suppressing inflammation and increasing nitric oxide bioavailability

Consumption of high-fat diet will lead to the formation of free radicals and hydroxyl products that can cause inflammation and an increase in NO inactivation, contributing to endothelial dysfunction and increasing risks in high blood pressure (Touyz 2004). In this study, rats fed with a high-fat diet showed an impaired vascular response. The high-fat diet might hinder endothelial relaxation in two ways; either by inactivating endothelium-derived NO or aggravating vascular smooth muscle contraction. The former is more likely compared to the latter. Our study demonstrated a decreased vascular response to Ach and SNP in the postmenopausal rats, indicating impairment of vasodilating and vasoconstricting capabilities of the vascular, leading to an increased in blood pressure (Han et al. 2018). This condition is in line with the reduced NO level. NO is an endothelium-derived relaxing factor (EDRF), and its synthesis occurs in the endothelial cells (Pirahanchi and Brown 2020). Therefore, damaged endothelial cells would affect the production of NO, causing the vascular response. The intake of high-fat diets, which are high in peroxides, might augment oxidative stress in the rats, later reducing the level of plasma NO. A similar observation was also noted in another study (Nurul-Iman et al. 2013). The NO released by the SNP, which action is not dependent on the presence of endothelium (Nurul-Iman et al. 2013), also does not manage to cause vasodilation possibly due to the effects of ROS on the released NO. The consumption of a high-fat diet also alters other vasoactive substances. It diminishes plasma prostacyclin and elevates plasma thromboxane levels (Siti et al. 2017).

Our result showed augmented vasorelaxation response to both Ach and SNP following treatment with GP and ATV compared to the PM rats. In addition to the increase in NO bioavailability, the increase in vasorelaxation response especially to SNP suggests that GP may increase the response of the VSMCs (vascular smooth muscle cells) to NO. This is further supported by the observation that the vasorelaxation responses are higher after treatment with GP compared to control (sham) animals (Ajay et al. 2003). We proposed that GP might also affect VSMCs directly and independently of their effects on endothelial cells as have been established in endothelium-denuded vessels. GP possibly induces the vasorelaxation through activation of BKCa channels or inhibition of Ca^2+^ channels. BKCa channels act as feedback inhibitors of Ca^2+^ influx, and hence, limiting constriction whereas Ca^2+^ channels cause contraction and thus precipitate vasoconstriction (Maaliki et al. 2019).

Inflammatory processes that mediate the pathogenesis of arteriosclerosis increase the vascular free radical formation that leads to endothelial dysfunction. In this study, the inflammatory biomarkers, such as IL-6, TNF- α, and CRP, increased in the postmenopausal group, consistent with another study (Liu et al. 2010a). Similarly, IL-6 expressed in animal and human atherosclerotic lesions promotes the expression of CRP expression in human coronary artery smooth muscle cells (Liu et al. 2010b). The GP ethanol extract has an anti-inflammation effect of reducing the inflammation triggered by free radicals produced from repeated heated palm oil (Jeon and Kwon 2016). It was shown in this study that GP extract supplementation could reduce the level of IL-6, TNF-α, and CRP in postmenopausal rats. A recent study suggested that steroid might be one class of anti-inflammatory compounds that suppressed the expression of inflammatory responses (Jarikasem et al. 2013) by inhibiting the vasodilation, increasing vascular permeability that occurred following an inflammatory insult, and decreasing leukocyte migration into the inflamed sites (Timotius and Rahayu 2020).

Adhesion molecules such as sVCAM-1 and sICAM-1 are also expressed by the stimulation of ROS (Cook-Mills et al. 2011), inflammatory cytokines such as TNF-α (Zhang et al. 2013), and oxidized LDL (oxLDL) on endothelial cells during inflammatory diseases (Khan et al. 1995). These adhesion molecules are crucial for activating endothelial dysfunction and are increased in the postmenopausal group. The adhesion molecules will attract leucocytes to the inflammation sites by adhering to the endothelial cells that line the blood vessels. The endothelial cells create cell surface adhesion molecules such as VCAM-1, causing monocytes and T-lymphocytes to adhere to the endothelium, and then moving underneath them by pressing between endothelial cells. Circulating monocytes and T-lymphocytes are drawn to the damaged area by chemoattractant cytokines (chemokines). The endothelial cells alter the shape, and the tight junctions between the endothelial cells detach, expanding the penetrability to fluid, lipids, and leukocytes. The LDL in the arterial wall is oxidized due to NO, macrophages, and some enzymes such as lipoxygenase. The migration of ox-LDL into the intima triggers the monocytes’ differentiation into macrophages to engulf the oxidized LDL. The lipid retained in the macrophages renders them more lipid-laden, and the macrophages are referred to as foam cells. The foam cell will undergo apoptosis and die, but the lipid will accumulate in the intima.

In the current study, GP extract supplementation at 500 mg/kg of body weight could regress sICAM-1 and sVCAM-1 expression in the postmenopausal rats. The expression of these molecules explains their responses to the inflammation stimuli, which trigger the interaction between the endothelium and blood cells during the atherosclerotic development (Fotis et al. 2012). Amidst the inflammation responses, the flavonoids in GP can impart anti- inflammatory effects by decreasing the expression of cell adhesion molecules (VCAM-1), intercellular adhesion molecule 1 (ICAM-1), E-selectin, pro-inflammatory and cytokines through the diminished nuclear translocation of nuclear factor kappa-light-chain-enhancer of activated B cells (NF-κB) transcription factor (Hsueh et al. 2016). NO acts as a vasodilation agent that relaxes vascular smooth muscle, inhibits platelet activation, and modulates migration growth of vascular smooth muscle. NO regulates genes that lead to the expression of adhesion molecules for monocytes, leading to an increase in oxidative stress that results in endothelial dysfunction. This observation is further supported by the higher vasorelaxation responses after treatment with GP compared to the control in the normal animals.

Supplementation with GP and ATV showed neither visible signs of toxicity nor mortality. These were showed by no changes on liver enzymes activities and indicates a good liver function (Hilaly et al. 2004). It suggested that supplementation of GP ethanol extract and ATV did not cause any harm or alter the hepatocytes structure of the rats.

## Conclusions

GP extract supplementation with a preferred dose of 500 mg/kg body weight improved the endothelial function by suppressing the inflammatory response, supporting the maintenance of blood pressure and vascular tone in postmenopausal condition.

## Supplementary Material

Supplemental Material

## Data Availability

The raw data supporting the conclusions of this article will be made available by the authors, without undue reservation to any qualified researcher.
