# Supplementary material for: Gynura procumbens ethanol extract improves vascular dysfunction by suppressing inflammation in postmenopausal rats fed a high-fat diet
Source: Pharm Biol. 2021 Sep 7;59(1):1201–13. doi: 10.1080/13880209.2021.1970199 (PMC8428271; doi:10.1080/13880209.2021.1970199)
Supplement: Supplemental Material [file IPHB_A_1970199_SM6215.docx]

S1: The HPLC chromatogram and LC-MS/MS data of *Gynura procumbens* extract


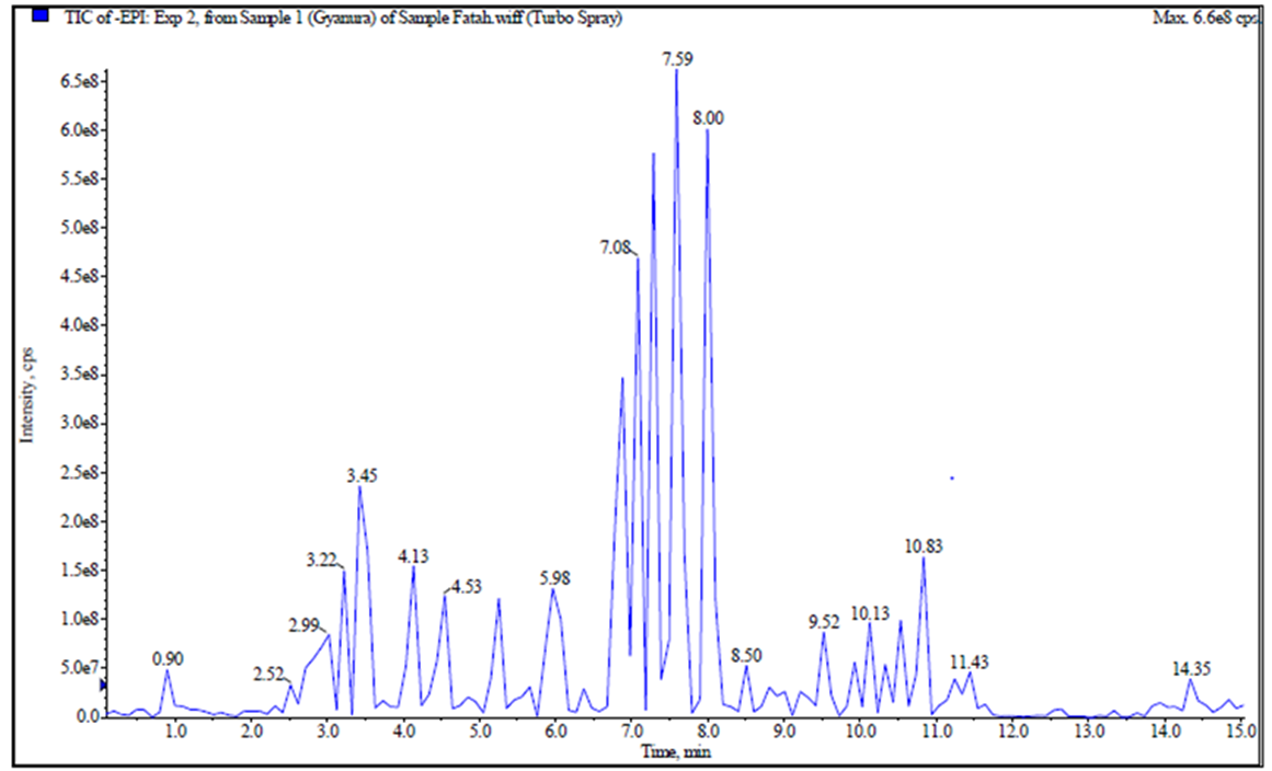


(1)

(4)

(6)

(14)

(16)

| **Identified compound** | **RT (min)** | **Molecular ion [M-H]^-^ (m/z)** |
| --- | --- | --- |
| 1. Unidentified | 0.9 | 244.990 |
| 2. Caffeic acid | - | 179.030 |
| 3. Trimethyl gallic acid glucuronide | - | 387.000 |
| 4. 5-O-(E)-caffeoyl-galactaric acid | 4.13 | 371.000 |
| 5. Chlorogenic acid | 4.33 | 609.520 |
| 6. Unidentified | 4.53 | 496.190 |
| 7. Rutin | 4.81 | 609.160 |
| 8. Quercertin | 4.91 | 300.923 |
| 9. Neochlorogenic acid | 4.99 | - |
| 10.Nicotiflorin | 5.02 | 593.000 |
| 11.Astragalin | 5.12 | 447.000 |
| 12.Kaempferol | 5.79 | 284.916 |
| 13.4-0 methyl gallic acid sulphate or 3-0 methyl gallic acid sulphate | - | 263.070 |
| 14.Oxooctadecanoic acid isomer | - | 298.170 |
| 15.Unidentified | 5.98 | - |
| 16.Genkwanin isomer | - | 572.290 |
| 17.Eriocitrin | - | 595.280 |
| 18.15,16-dihydroxy-9Z,12Z-octadecadienoic acid | 7.08 | 311.200 |
| 19.Unidentified | 7.59 | - |
| 20.Oxooctadecanoic acid derivative | - | 312.190 |
| 21.Oxooctadecanoic acid | 9.52 | 297.300 |
| 22.Unidentified | 10.83 | 817.000 |


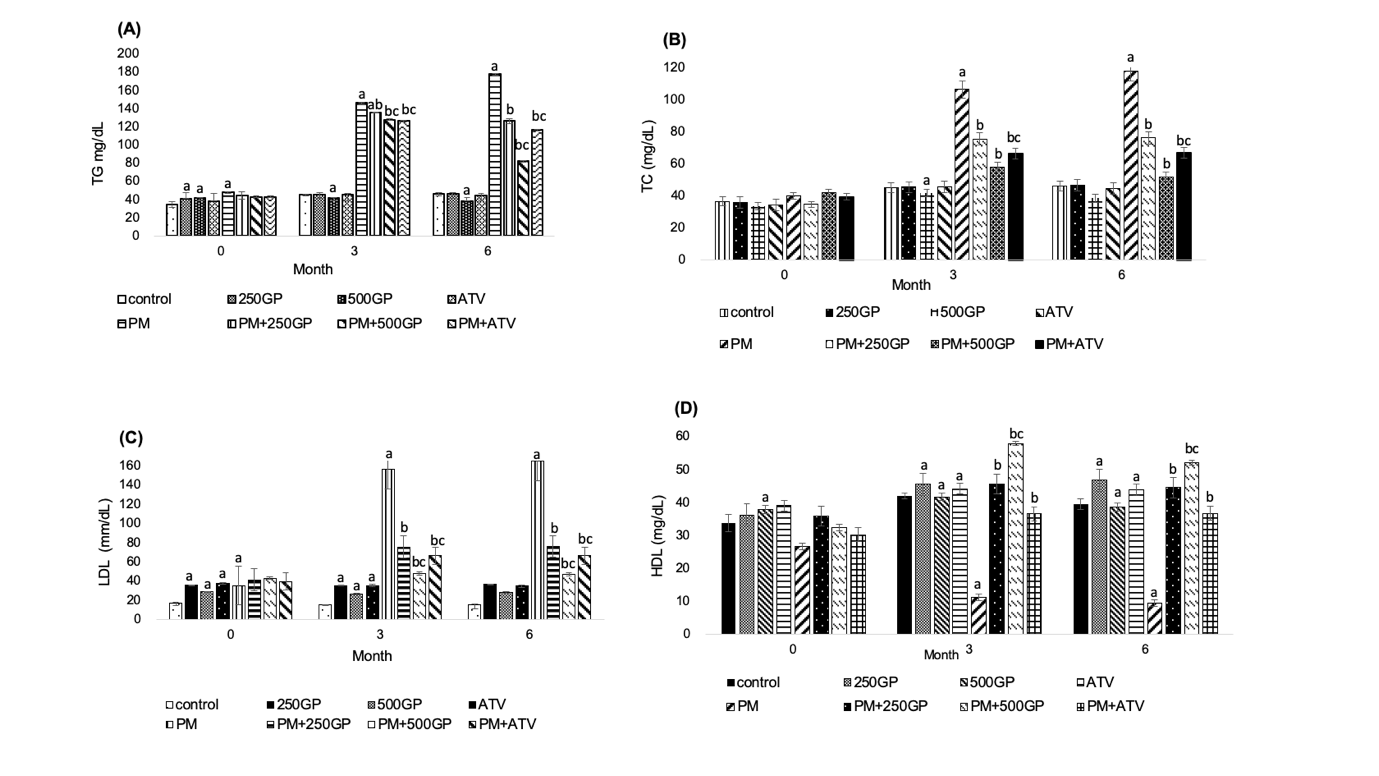


S2: Effects of *Gynura procumbens* extract supplementation on serum lipid profiles in sham and postmenopausal (PM) groups at 0, 3, and 6 months of study.
